# Interaction of High-Molecular Weight Fucoidan from *Laminaria hyperborea* with Natural Functions of the Retinal Pigment Epithelium

**DOI:** 10.3390/ijms24032232

**Published:** 2023-01-23

**Authors:** Philipp Dörschmann, Georg Kopplin, Johann Roider, Alexa Klettner

**Affiliations:** 1 Department of Ophthalmology, University Medical Center, University of Kiel, Arnold-Heller-Str. 3, Haus 25, 24105 Kiel, Germany; 2 Alginor ASA, Haraldsgata 162, 5525 Haugesund, Norway

**Keywords:** sulfated fucan, age-related macular degeneration, wound healing, retinal pigment epithelium-specific 65 kDa protein (RPE65), vascular endothelial growth factor (VEGF), inflammation, phagocytosis, gene expression, Raman, Fourier-transform infrared spectroscopy (FTIR)

## Abstract

Fucoidans are polysaccharides and constituents of cell walls of brown algae such as *Laminaria hyperborea* (LH). They exhibit promising effects regarding age-related macular degeneration (AMD). However, the safety of this compound needs to be assured. The focus of this study lies on influences of an LH fucoidan on the retinal pigment epithelium (RPE). The high-molecular weight LH fucoidan Fuc1 was applied to primary porcine RPE cells, and a tetrazolium (MTT) cell viability assay was conducted. Further tests included a scratch assay to measure wound healing, Western blotting to measure expression of retinal pigment epithelium-specific 65 kDa protein (RPE65), as well as immunofluorescence to measure uptake of opsonized fluorescence beads into RPE cells. Lipopolysaccharide was used to proinflammatorily activate the RPE, and interleukin 6 (IL-6) and interleukin 8 (IL-8) secretion was measured. RPE/choroid cultures were used to assess vascular endothelial growth factor (VEGF) secretion. Real-time polymerase chain reaction (real-time PCR) was performed to detect the gene expression of 91 different genes in a specific porcine RPE gene array. Fuc1 slightly reduced wound healing, but did not influence cell viability, phagocytosis or RPE65 expression. Fuc1 lowered IL-6, IL-8 and VEGF secretion. Furthermore, Fuc1 did not change tested RPE genes. In conclusion, Fuc1 does not impair RPE cellular functions and shows antiangiogenic and anti-inflammatory activities, which indicates its safety and strengthens its suitability concerning ocular diseases.

## 1. Introduction

Sulfated fucans are marine polysaccharides predominantly composed of l-fucose, and a fucose backbone structure. They are also known as fucose-containing sulfated polysaccharides (FCSP) and the term fucoidan has been used historically for all FCSP before the analysis of fine structure allowed for further classification. Fucoidans as protective mucilage are mostly found in brown seaweed cell walls and partially in marine invertebrates. Their natural functions consist of stability of the cell wall as it links cellulose and alginates, and protection against dehydration and environmental factors such as pathogens, osmotic pressure and tides [1,2]. The structural properties of fucoidans, such as sugar composition, molecular weight, substitution with sulfate groups and acetyl groups, as well as backbone structure and branching, strongly depend on the brown seaweed species as well as seasonal and regional variation and extraction methods. These render fucoidans as a very heterogeneous molecule group. Their large abundance in brown algae cell walls proposes potential for a multitude of health products, food supplements and medications, derived from a sustainable resource [3]. A large variety of bioactive effects has been reported for fucoidans, such as anticancer, antidiabetic, anti-inflammatory and anticoagulative properties [4,5,6,7]. Fucoidans are also interesting substances for possible treatment of ocular diseases such as age-related macular degeneration (AMD) [8,9] and uveal melanoma [10,11,12]. The bioactive properties of fucoidans are directly linked to their structural characteristics; therefore, a structural elucidation is necessary to uncover structure–function relationships [8,9,10,11,12,13,14,15,16].

AMD is the main cause of blindness in the elderly in industrialized nations. AMD presents itself in an early form, which is not symptomatic for the patient, and two different late forms. On the tissue level, AMD progresses into the degeneration of the retinal pigment epithelium (RPE), followed by a degeneration of photoreceptors [17]. In dry AMD, accumulation of oxidized lipid or protein molecules and immune complexes may lead to an atrophic degeneration of the retinal pigment epithelium (RPE) and photoreceptors, while in wet AMD neovascular vessels grow from the underlying choroid beneath and into the retina, causing rapid tissue deterioration [17]. The most important factors are oxidative stress and a high secretion of vascular endothelial growth factor (VEGF) secretion, with the RPE as the main source [17,18]. Only for wet AMD, treatment options (anti-VEGF therapeutics) are available which must be applicated regularly into the eye [19]. Fucoidans are described to be protective against oxidative stress and to inhibit VEGF [10,11,12,13,14,20]. In addition, they also can exhibit antitumorigenic activities [10,12]. These biological activities of fucoidans improve with the higher grade of purity, measured as fucose content, excluding the use of crude fucoidans [14,15].

*Laminaria hyperborea* (LH) is a large brown seaweed of the family Laminariaceae (kelp). It is also known as cuvie or tangle and grows in the northeast Atlantic Ocean near Scandinavia. LH is a sustainable source of fucoidans. We have previously tested several algae species sources and their fucoidans for beneficial effects in ocular cells. Fucoidans from LH and *Saccharina latissima* showed the most promising effects concerning AMD [11,13,14]. The LH fucoidan (Fuc1) contains a very high amount of fucose (97%), which renders this agent as highly pure fucoidan [5,13]. The degree of sulfation is 1.7, molecular weight is 1548.6 kDa and structural analysis revealed a random coil structure [13]. Toxicity tests showed that short-term cell viability was not affected for ARPE-19 (human, immortal RPE cell line) or primary porcine RPE cells [13]. In addition, this LH fucoidan had slightly protective effects against hydrogen peroxide in the uveal melanoma cell line OMM-1 [13]. Of the tested fucoidans, it displayed the strongest effect in inhibiting VEGF in ARPE-19 and the primary RPE [13]. Furthermore, this fucoidan showed protective effects against ferroptosis and oxidative stress by slightly increasing cell viability, again after erastin treatment [21], and increasing glutathione peroxidase 4 (GPX4) expression [21], rendering this fucoidan as antioxidative. All these biological activities mark this fucoidan as a possible new drug for the treatment of AMD [13,20]. The potential of this fucoidan could be used for prevention of the progression of AMD from the early to the late forms of this disease, interfering with the pathomechanisms before vision loss manifests in patients [8]. For such preventive use it is vital that this compound does not interfere with the viability or the function of the RPE.

The RPE is a single layer epithelial cell type with distinct “cobblestone” morphology. It is the main “housekeeper” for the maintenance of the photoreceptors and also the most affected cell type in AMD [22,23]. It provides the photoreceptors with nutrients and discards their waste material. It is involved in the recycling of visual pigments, expressing the key molecule RPE-specific 65 kDa protein (RPE65), and devours photoreceptor outer segments, taken up by phagocytosis [22]. In addition to standard tests for cell viability, proliferation and toxicity, it is of importance to detect marker proteins and genes that represent the main functions of the RPE, to exclude negative impacts of fucoidan. Genes that are substantial for the normal function of the RPE should not be negatively influenced [24]. Further, the differentiation should be maintained and a mesenchymal transition should be excluded, so that the RPE can sustain its cellular functions. Another task of the RPE is the downregulation of the immune response in the retina. Furthermore, it acts as a sentinel for danger signals and expresses toll-like receptors (TLR) to detect pathogens and danger-associated molecular patterns [25]. TLR4 activation and proinflammatory stimulation via lipopolysaccharide can lead to proinflammatory secretion of cytokines such as interleukin 6 (IL-6), interleukin 8 (IL-8) and tumor necrosis factor α (TNF) [26].

This study is a continuation of a series of research works to determine if LH fucoidans are suitable for future application in medicine concerning ocular diseases, especially AMD. The main aim of this study is to test whether this promising, pure, sustainable high-molecular weight LH fucoidan is suitable for further research concerning ocular diseases by testing its effects on RPE viability, morphology, cellular functions (wound healing, phagocytosis), cytokine secretion and gene expression. The exclusion of negative effects in RPE paves the way for further testing in animal models. Furthermore, this study provides a deeper insight on the molecular structure and composition of this fucoidan extract.

## 2. Results

### 2.1. Effects on Mid-Term Morphology and Viability

Cell viability and morphology are important indicators for possible stimulating or toxic effects of biological test substances. A former study already showed no relevant effects after stimulation of RPE with the LH fucoidan from Alginor ASA (1548.6 kDa, Fuc1) for 24 h [13].

**Figure 1:**
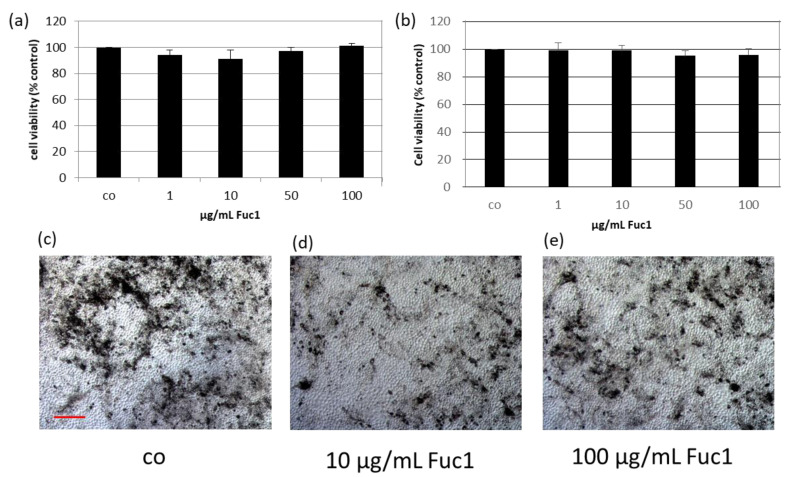
The effects on cell viability of primary retinal pigment epithelium cells (RPE) were determined with tetrazolium (MTT) assay after stimulation with 1–100 µg/mL high-molecular weight *Laminaria hyperborea* (LH) fucoidan Fuc1 for three (**a**) and seven days (**b**). Mean and standard deviation is depicted in percent relative to untreated control (set to 100%). Data was normally distributed (Shapiro–Wilk test). Significances were determined with analysis of variance (ANOVA) and post-hoc Dunnett’s multiple comparison test. No significant effects were determined. Further, bright field images of the RPE cell layer were taken after seven days (100× magnification, red scale bar = 100 µm). The morphology was qualitatively investigated and not influenced by Fuc1 (**c**–**e**). co = control; *n* = 5 (number of independent experiments).

The effects of Fuc1 on cell viability of primary RPE cells were detected with a tetrazolium (MTT) assay. For that, the ocular cells were treated for three (Figure 1a) and seven (Figure 1b) days with the LH fucoidan Fuc1 in concentrations of 1, 10, 50 and 100 µg/mL. Cell viabilities were transformed in relative percent compared to untreated control, which was set to 100%. No significant effects on cell viability were determined. In addition, no effect on morphology, as assessed by light microscopy, was seen after seven days of treatment (Figure 1c–e). The RPE cells were tightly connected and in a hexagonal, monolayer (“cobblestone”) pattern. No dedifferentiation or mesenchymal transition could be found.

### 2.2. Effects on Wound Healing

**Figure 2:**
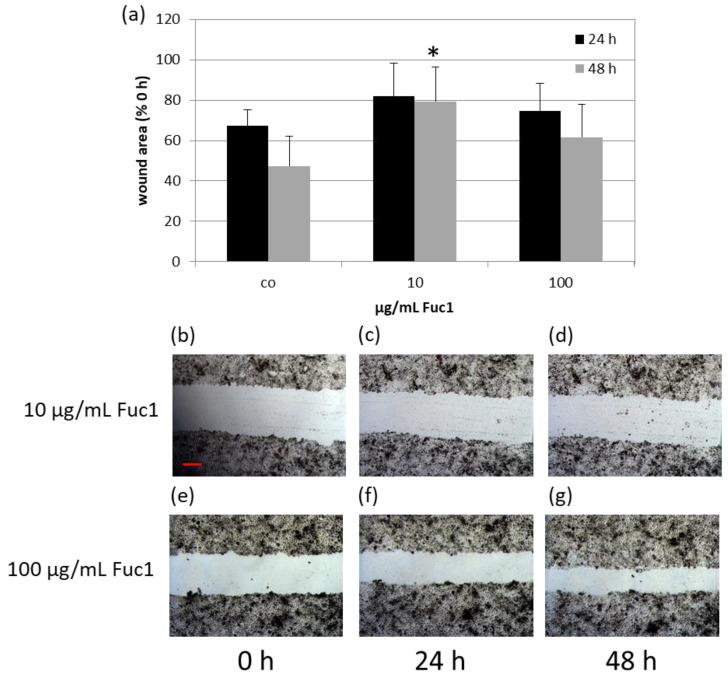
Primary RPE cells were stimulated with 10 or 100 µg/mL Fuc1, respectively, for three days. After this, a wound was applied into the cell layer with a pipette tip, photos were captured with a light microscope camera and the area was measured with the software AxioVision 0, 24, and 48 h post-scratch (10 µg/mL Fuc1 (**b**–**d**); 100 µg/mL Fuc1 (**e**–**g**)). The mean values and standard deviation are depicted as relative percent values to the initial scratch area (**a**). Photos of initial scratch as well as 24, and 48 h post-scratch show the wound after stimulation with 10 or 100 µg/mL Fuc1 (50× magnification, red scale bar = 100 µm). Data were normally distributed (Shapiro–Wilk test). Significances were determined with analysis of variance (ANOVA) and post-hoc Dunnett’s multiple comparison test. co = control; h = hours; * *p* ≤ 0.05 against control of matching post-scratch time; *n* = 5 (number of independent experiments).

To investigate the wound healing ability of RPE cells, a scratch assay was performed after stimulating the cells for three days with 10 and 100 µg/mL Fuc1, respectively. For that, a scratch with a sterile pipette tip was applied into the cell layer and photos were taken immediately post-scratch and after 24 and 48 h (Figure 2b–d for 10 µg/mL Fuc1 and Figure 2e–g for 100 µg/mL Fuc1) with an inverse light microscope camera. The wound area was measured with the microscope software AxioVision. After setting the values in relation to the initial scratch area, the data give an indication about the regeneration and migration ability of the RPE cells (Figure 2a). No significant effects could be detected with the exception of 10 µg/mL Fuc1, 48 h after applying the wound. In this case, delayed wound regeneration could be determined with 79% ± 17% wound area after Fuc1 treatment compared to 47% ± 15% wound area of the untreated control (*p* = 0.013). For visualization, exemplary scratch photos with delayed wound healing after 0, 24 and 48 h can be seen in Figure 2b–d (treatment with 10 µg/mL Fuc1).

### 2.3. Effects on RPE65 Expression

**Figure 3:**
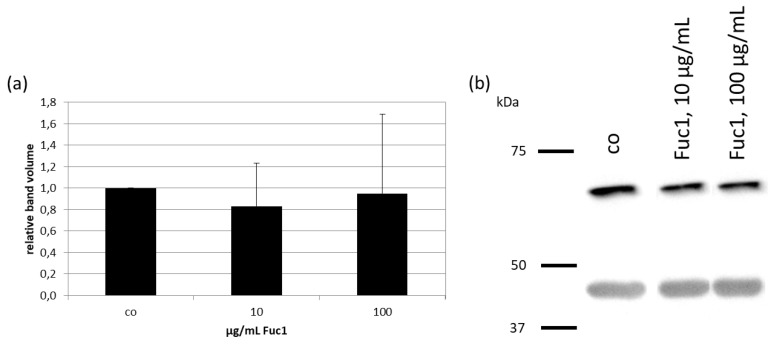
Primary RPE cells were stimulated with 10 or 100 µg/mL Fuc1 for three days. Cell lysates were made and applied in Western blot to detect RPE-specific 65 kDa protein (RPE65) expression in relation to β-Actin (48 kDa). Exemplary blot is depicted (**b**). Band volumes were densiometrically evaluated with TotalLab, RPE65 volumes normalized with data of β-actin expression and calculated relative to control (set to 1.0, (**a**)). Data were normally distributed (Shapiro–Wilk test). Significances were determined with analysis of variance (ANOVA) and post-hoc Dunnett’s multiple comparison test. No significant effects were determined. co = control, kDa = kilodalton and *n* = 4 (number of independent experiments).

After treating RPE cells with 10 or 100 µg/mL Fuc1 for three days, cell lysates were made. Protein level was determined and proteins were applied for gel electrophoresis and wet tank blotting (Western blotting) for the detection of RPE65 (example blot in Figure 3b). Further, expression of β-Actin as performance control was determined. The band volumes were evaluated with TotalLab software, normalized with the data of β-Actin, and put in relation to the untreated control, set to 1.0. No significant changes in protein expression level of RPE65 could be detected (Figure 3a), indicating Fuc1 as not interfering with visual pigment recycling.

### 2.4. Effects on Phagocytosis

**Figure 4:**
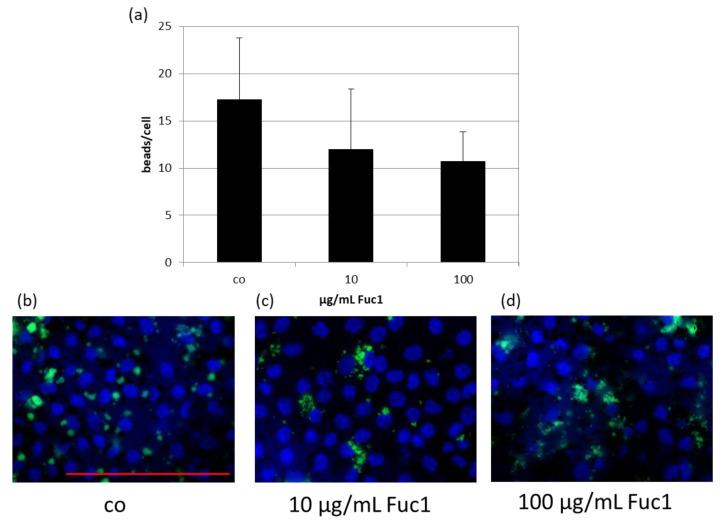
Primary RPE cells were stimulated with 10 or 100 µg/mL Fuc1 for three days. To measure phagocytic ability, fluorescent beads opsonized with photoreceptor outer segments were added to the cells (green dots). Cell were fixated with paraformaldehyde and treated with Hoechst to mark cell nuclei (blue circles). Photos were taken with an immunofluorescence microscope ((**b**–**d**); 640× magnification, red scale bar = 100 µm). Beads and nuclei were counted with Fiji Software (ImageJ2). Number of beads was put in relation to the cell nuclei (**a**). Data were normally distributed (Shapiro–Wilk test). Significances were determined with analysis of variance (ANOVA) and post-hoc Dunnett’s multiple comparison test. No significant effects were determined. co = control; *n* = 5 (number of independent experiments).

RPE cells on collagen I-coated cover slips were treated with 10 or 100 µg/mL Fuc1 for three days, after which a phagocytosis assay was performed. The uptake of latex beads (green), conjugated with photo receptor outer segments, was counted via Fiji (Image J) on fluorescence images (Figure 4b–d and put in relation to the number of cell nuclei (blue) (Figure 4a). No significant changes of the bead to cell relation number were detected, indicating that this LH fucoidan does not interfere with phagocytic ability of the cells.

### 2.5. Effects on VEGF Secretion

**Figure 5:**
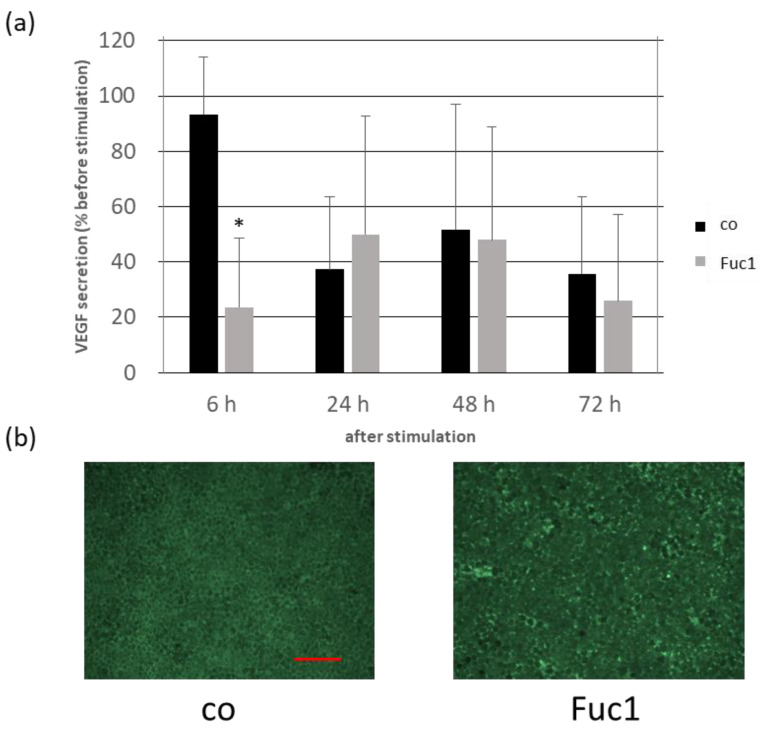
Organ cultures consisting of primary porcine RPE and choroid were clamped into rings and put into perfusion chambers with a steady media flow Two chambers were used in parallel; one of them was stimulated with 50 µg/mL Fuc1 for 72 h. Samples were taken before and after six, 24, 48 and 72 h of stimulation. Depicted is the vascular endothelial growth factor (VEGF) secretion in percent of the initial amount before stimulating (**a**). After 72 h the tissues were stained with calcein to label vital cells in green ((**b**): 100× magnification, red scale = 100 µm). Activated cells are light green; death cells are black. Data were normally distributed (Shapiro–Wilk test). Significances were determined with analysis of variance (ANOVA) and post-hoc Dunnett’s multiple comparison test. * *p* ≤ 0.05 compared to control, h = hours and *n* = 3 (number of independent experiments).

We already demonstrated in a previous study that 50 µg/mL Fuc1 was most efficient in reducing VEGF secretion of primary porcine RPE cultures after three days of stimulation [13]. We wanted to check and confirm whether VEGF secretion is influenced in organ cultures consisting of primary porcine RPE and choroid embedded in a perfusion culture system with steady state media flow. Two chambers with six rings of RPE/choroid tissue were used each time; one of them was stimulated with 50 µg/mL Fuc1 for 72 h. Samples were taken before and after 6, 24, 48 and 72 h and analyzed with a VEGF enzyme-linked immunosorbent assay (ELISA, Figure 5a). After this, tissue was removed and stained with calcein to label vital cells in green. Fluorescence images were taken and exemplarily depicted (Figure 5b). Again, we could demonstrate the VEGF-inhibiting effect of Fuc1; however, in a different time frame. In the case of this organ culture, Fuc1 reduced VEGF secretion after six hours to 24 ± 30% compared to the control with 93 ± 26% (*p* = 0.039). Calcein staining revealed no toxic effect of the fucoidan to the tissue but some cells were activated (light green), indicating a small metabolic activating effect.

### 2.6. Effects on Gene Expression

**Figure 6:**
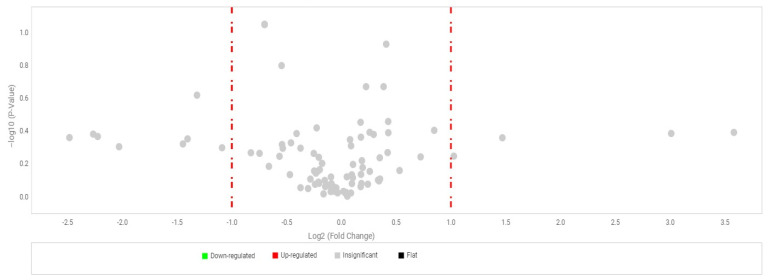
Primary RPE cells were stimulated with 50 µg/mL Fuc1, RNA was isolated for cDNA generation, and real-time polymerase chain reaction with TaqMan™ gene arrays was conducted. Here, 91 different porcine genes (Appendix A) were tested and no significant changes could be determined. Genes for glyceraldehyde-3-phosphate dehydrogenase (*GAPDH*), actin beta (*ACTB*) and glucuronidase beta (*GUSB*) were used as endogenous controls for normalization. This figure shows a volcano plot. Dots to the left and right of the red line represent genes which are two times up- or down-regulated, but the grey coloring represents that they were not significant. Evaluation was performed with cloud-based relative quantification software (Thermo Fisher Connect™, Thermo Fisher Scientific, Inc., Waltham, MA, USA). Student’s *t*-test within the software was used for significance calculation. *n* = 3 (number of independent experiments).

RPE cells were treated with 50 µg/mL Fuc1, and untreated RPE cells were cultivated in parallel. This concentration showed the most effective activity concerning VEGF inhibition in a previous study [13]. After an incubation time of three days the RNA was isolated and transformed to cDNA, which was used for a real-time polymerase chain reaction (real-time PCR). TaqMan™ gene arrays were used which were custom-made and designed for porcine RPE genes, including 91 different porcine RPE- relevant gene primers (list of genes in Appendix A). The cloud-based Software Thermo Fisher Connect™ was used for evaluation. Genes for glyceraldehyde-3-phosphate dehydrogenase (*GAPDH*), actin beta (*ACTB*) and glucuronidase beta (*GUSB*) were used as endogenous controls for normalization. No relevant up- or down-regulation or significant change in gene expression was seen (volcano plot in Figure 6, relative-fold gene expression level RQ (=2^−ΔΔCT^) in Appendix A). The gene expression of *CCL11* (CC-chemokine ligand 11), *CRP* (C-reactive protein), *CX3CR1* (CX3C chemokine receptor 1) and *LEP* (leptin) tended to be reduced. This suggests that Fuc1 may have slight effects on inflammation-, complement system- and lipid metabolism-relevant genes. Overall, fucoidan Fuc1 does not interfere with expression of typical important RPE genes. For example, the gene for RPE65 was not influenced, as previously shown on protein level (Section 2.3).

### 2.7. Secretion of Proinflammatory Cytokines

**Figure 7:**
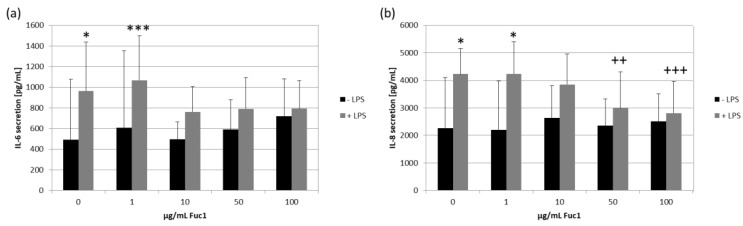
Primary RPE cells were stimulated with 1, 10, 50 and 100 µg/mL Fuc1 and/or 1 µg/mL lipopolysaccharide (LPS) for seven days and supernatants were taken. These were analyzed with ELISA for interleukin 6 (IL-6, (**a**)) and interleukin 8 (IL-8, (**b**)) secretion. Data were normally distributed (Shapiro–Wilk test). Significances were determined with analysis of variance (ANOVA) and post-hoc Dunnett’s multiple comparison test. * *p* ≤ 0.05; *** *p* ≤ 0.001 compared to 0 µg/mL Fuc1 without LPS; ++ *p* ≤ 0.01 and +++ *p* ≤ 0.001 compared to 0 µg/mL Fuc1 with LPS; *n* = 8 (number of independent experiments).

For inflammatory cytokine testing we used confluent primary RPE cells, which were stimulated with 1 µg/mL lipopolysaccharide (LPS) and/or 1–100 µg/mL Fuc1 for seven days. Supernatants were collected for three days. The MTT assay was conducted to measure cell viability, and, in all cases, it was not affected. Supernatants were analyzed for IL-6 (Figure 7a), IL-8 (Figure 7b) and TNF secretion with ELISA. No relevant TNF secretion was detected after stimulating with LPS; in addition, the fucoidan alone did not increase TNF secretion at all. Regarding IL-6 secretion, LPS could significantly increase it up to 962 ± 475 pg/mL (*p* = 0.013). Fuc1 did not increase IL-6 on its own. Remarkably, the Fuc1 concentration from 10–100 µg/mL could lower IL-6 secretion in the presence of LPS as it showed no significant effects anymore. LPS increased IL-8 secretion up to 4227 ± 926 pg/mL (*p* = 0.043). Fuc1 did not increase IL-8 secretion on its own. Furthermore, it significantly decreased the LPS-activated IL-8 amount at concentrations of 50 and 100 µg/mL down to 2992 ± 1311 pg/mL (*p* = 0.004) and to 2809 ± 1146 pg/mL (*p* = 0.001), respectively. These data indicate an anti-inflammatory effect of Fuc1.

### 2.8. Characterization through Vibrational Spectroscopy—FTIR and Raman

**Figure 8:**
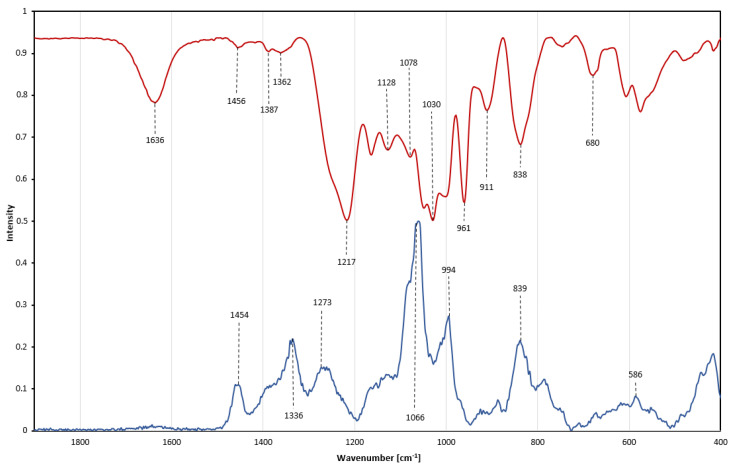
Vibrational spectra of Fuc1. Overlay of Raman spectrum (blue line) and Fourier−transform infrared spectrum (red line, displayed as absorbance) from 500 to 1800 cm^−1^.

Raman and infrared (IR) spectroscopy are complementary methods. Vibration bands in both methods were assigned to different atom bonds and groups, further characterizing the molecular structure and composition as well as helping to detect impurities [27]. The Raman and Fourier-transform infrared (FTIR) spectra of fucoidan Fuc1 are presented in Figure 8 and Table 1. Characteristic vibration bands for fucoidan are found at 1454 cm^−1^ and were assigned to symmetric CH_3_ deformations from the fucose 6C methyl group [28].

Assessment of sulfation: A characteristic peak in the Raman spectrum is the intense band at 1066 cm^−1^, which originates from symmetric sulfate ester stretching vibrations [28,29,30]. This O=S=O stretching band is commonly used to estimate the degree of polysaccharide sulfation, due to its high intensity, even though a general overlap of C-O and C-C pyranoid ring vibrations between 970–1200 cm^−1^ occurs, as well as O-C-O stretching vibrations originating from glycosidic linkages [31,32,33]. In the infrared spectrum a corresponding band is located at 1217 cm^−1^ resulting from anti-symmetrical S=O vibration, with a shoulder band at 1257 cm^−1^, additionally attributed to S=O stretching movements [34]. A small IR peak is found at 838 cm^−1^, originating from C-O-S deformations in the C-2 position of the fucose ring. This IR peak is accompanied by a shoulder band at 820 cm^−1^ attributed to an α-anomeric C-H deformation [27,28,29,35]. A further characteristic sulfate band is displayed in the Raman spectrum at 839 cm^−1^ and assigned to a sulfate group in the C-4 position of the fucose ring [5,36]. Although the intensity of this band is lower than for the main peak at 1066 cm^−1^, the band on the other hand has also less overlap with other bands within the carbohydrate [5]. The envelope around 586 cm^−1^ in the Raman spectrum reflects a variety of different symmetric and antisymmetric internal SO_4_ vibrations but also corresponds to a variety of pyranoid ring vibrations [35,37,38]. By combining FTIR and Raman, further insight into sulfate position and peak assignment on a previously well-characterized fucoidan was achieved.

Purity assessment: Most fucoidans exhibit strong peaks between 1650–1800 cm^−1^, which are related to C=O vibrations. The absence of such signals verifies the absence of uronic acids as well as any acetylation within the fucoidan chain [39]. The IR spectrum shows a band at 1636 cm^−1^, which is also correlated with double bonds such as C=O or C=C [40]. However, those double bonds produce a very strong IR band between 1600–1800 cm^−1^; even trace amounts of alginate would still be detectable [33]. The presence of polyphenolic compounds would be indicated by very strong aromatic ring vibrations around 1600 cm^−1^ in the Raman spectrum, as well as characteristic weak bands around 3000 cm^−1^. No polyphenolic bands have been observed [41]. IR and Raman spectroscopy allows for a quick qualitative non-quantitative assessment of impurities within fucoidan samples.

## 3. Discussion

The aim of this study was to evaluate the impacts of the high-molecular weight LH fucoidan Fuc1 on primary, porcine RPE cells with focus on important cellular functions. In addition, the chemical composition and structure was investigated in more detail. Regarding a possible treatment or prevention of eye diseases it is important that the main functions of the RPE are not impaired, so that this cell type can still support the photoreceptors and contribute to accurate vision.

A previous study already showed that this well-characterized high-molecular weight LH fucoidan exhibits a stronger influence on different biological activities which are of interest in AMD prevention in comparison to its smaller variants [13]. The biggest LH fucoidan (1548.6 kDa) exhibited the most effective VEGF inhibition in the human RPE cell line ARPE-19 and in primary RPE cells [13]. It was not antiproliferative in short-term stimulations for both cell types and it achieved slightly antioxidative effects against hydrogen peroxide in OMM-1 [13]. It was also described that high concentrations of this fucoidan can inhibit coagulation, complement proteins and cytokines such as PDGF-BB (platelet-derived growth factor BB), CCL5 (chemokine (C-C motif) ligand 5) and CXCL10 (C-X-C motif chemokine ligand 10), whilst it can activate CCL2 (chemokine (C-C motif) ligand 2), and also stated that the effects are highly dependent on degree of sulfation, molecular weight and concentration of the fucoidan [5]. It was also shown that Fuc1 fucoidan can exhibit antioxidative effects on the glutathione ferroptose pathway as it was protective against erastin-induced cell death and increased GPX4 expression [21]. Other biological effects of LH fucoidans are, to our knowledge, not investigated so far. For a potential application in the eye, viability, morphology or regeneration ability of the cells should not be affected by this fucoidan. Furthermore, the phagocytosis of used photoreceptor outer segments and the renewal of the visual pigment through RPE65 should not be influenced by the extract.

The first step to further investigate Fuc1 was to determine its effects on cell viability with prolonged stimulation times. Short-term stimulation indicated no significant effects on cell vitality [13]. Similarly, in this study a mid-term stimulation time showed no relevant effects on cell viability, which showed that the overall metabolism of the RPE cells is not affected in the course of one week. Further, the light microscopy offered no disturbance in the morphology of the RPE cells. These results are supported by Bittkau et al., 2019, which stated that fucoidans cannot be considered as antiproliferative or toxic in general [12]. However, these effects depend on the exact combination of cellular model system and the distinct brown algae extract. For example, *Dictyosiphon foeniculaceus* reduces the viability of the tumor cell line OMM-1 [10,12]. Of note, the effect could be related to the purity of the extract, which had a rather low fucose content [16], suggesting other agents such as proteins can interfere with the viability of the cells. In contrast, the extract of this study is highly pure and consists of 97% fucose [13], supporting its beneficial biological activities.

Furthermore, the regeneration and migration ability of the ocular cells was not influenced, which was determined with a wound healing assay, with one exception. Here the lower concentration of Fuc1 showed a slightly significant reduced regenerative ability of the cells 48 h post-scratch. This effect was demonstrated before by Dithmer et al., 2014 [20], who tested commercial *Fucus vesiculosus* fucoidan from Sigma Aldrich. It also reduced wound healing ability, but at a higher concentration of 100 µg/mL and at an earlier time point (24 h) [20]. In addition, Rower et al. 2019 showed that crude fucoidan extract from *Fucus distichus* subsp. *Evanescens* can reduce wound healing ability [15]. Therefore, the effect on wound healing ability seems to be inherent to fucoidans. It should be noted, however, that the effect seen with this fucoidan, despite its statistical significance, seems to be transient (higher concentration showed no effect) and most likely of little biological consequence, especially considering that the RPE in situ is a post-mitotic cell [42].

RPE65 is a key molecule in the visual cycle and is expressed exclusively by the RPE [43,44]. In the enzymatic process during phototransduction, all-trans-retinyl esters are transformed to 11-cis-retinol, which is important for the regeneration of rhodopsin [43]. This process is vital for the process of vision. Mutations and changes in the protein sequence of this protein can lead to blinding diseases such as retinitis pigmentosa and Leber’s congenital amaurosis [43]. Therefore, a possible drug or food supplement should not interfere with the expression of RPE65. This study showed that Fuc1 does not significantly influence the expression of RPE65.

Phagocytosis of used photoreceptor outer segments is an important task of the RPE and vital to uphold vision [22]. Royal College of Surgeons rats were not able to exhibit the natural phagocytosis functions of the RPE cells which inhibit the recycling of shed photo receptor outer segments, leading to an accumulation of membrane debris in the subretinal space and a consequent degeneration of photoreceptors [45]. The data of this study show no significant impact on phagocytosis. This is in accordance with a previous study, where short-term treatment with Sigma-Aldrich fucoidan from *Fucus vesiculosus* showed no effects on phagocytosis functions of RPE [20]. However, another study investigating crude *Fucus distichus* subs. *Evanescens* showed that it lowered the phagocytosis ability of RPE after mid-term stimulation [15], which indicates the importance of checking the influence of the specific fucoidan and the connection to its chemical properties and purity.

VEGF secretion is an important task of the RPE to fenestrate blood vessel endothelial cells to support the photoreceptors [22]. If this secretion is pathologically increased, it may cause wet AMD with an uncontrolled growth of blood vessels under and into the retina as well as leakage [17]. We demonstrated before that Fuc1 can reduce VEGF secretion in a cell model system of primary porcine RPE and the human ARPE-19 cell line. With this study we wanted to test the effects on RPE/choroid organ cultures, one step closer to the real in vivo system. VEGF secretion was diminished again, but with a different time frame, showing significance already after six hours of stimulation. This effect’s significance was transient and not found for later time points (until 72 h), which is in contrast to the finding with RPE cell cultures [13]. This indicates how important the biological model system is. The VEGF-inhibiting effect cannot be explained by a simple scavenging effect because the fucoidan was constantly given to the tissue by the perfusion system. Rather, it could be explained by an initial VEGF receptor-inhibiting effect which may be counteracted by an adaption of the tissue to the fucoidan. It is know that fucoidans can bind VEGF receptor 2 and thereby may inhibit the VEGF self-regulating mechanisms via the Erk signaling pathway [46,47]. Another explanation for the short-term effect could be found in the experimental system, as the data show that VEGF was reduced over time in untreated organ cultures, which might have simply masked any effect of the fucoidan on VEGF. In another study fucoidan from *Fucus vesiculosus* reduced the VEGF amount of RPE/choroid explants not after six, but after 24 and 72 h. Here, the fucoidan was just initially given to the system and not in the constant media flow [20]. The different effects could be explained by the different methodological approach or the different fucoidan. Of note, we used a high-molecular weight fucoidan which shows more effective bioactivities regarding pathological AMD factors such as VEGF inhibition and oxidative stress protection [13], whilst smaller fucoidans are rather proangiogenic [48]. Another study showed also the beneficial effects of a high-molecular weight fucoidan which were antioxidative, anti-inflammatory, antihyperglycemic and anticoagulant by showing radical scavenging activity, inhibition of cyclooxygenase-2, hyaluronidase, mitogen-activated protein kinase p38 and dipeptidyl peptidase-IV as well as increased thrombin time [49].

Currently, the treatment options for AMD are limited, and only the wet form can be treated with anti-VEGF therapeutics [19], which have to be injected regularly into the eye. Moreover, the initial beneficial effect is often lost after some years [50]. Furthermore, VEGF has physiological functions and is also important for the fenestration of the blood vessels [22]. At the time of this study, four existing anti-VEGF therapeutics are available: bevacizumab, ranibizumab, aflibercept and the new brolucizumab. They all neutralize VEGF by interfering with its binding to its receptors to halt vessel leakage and growth [51]. Our aim was to use the fucoidan as a supportive or preventing agent, as due to its antiangiogenic, antioxidative and anti-inflammatory activities it possibly could prevent the AMD progression of the early non-symptomatic forms to the later dry or wet forms. It could be used as a dietary supplement to prevent the necessity of invasive treatment with the regular anti-VEGF drugs. In rats it was shown that orally fed fucoidan can be found in tissues such as kidney or liver with a long absorption and blood circulation time [52]. Furthermore, our tested fucoidans do not completely reduce VEGF secretion or expression, so the natural beneficial effects of VEGF can still be maintained. In addition, how fucoidan may act as adjunctive therapy for the four other anti-VEGF therapeutics can be further investigated. In a former study we already showed that fucoidans have an additional VEGF expression-lowering effect in RPE cells when given simultaneously with bevacizumab [20]. Moreover, fucoidans in general are non-toxic for human healthy cells [12,13], but further studies on animals should be made concerning long-term effects and metabolic activities.

The last step In investigating the effects on cellular functions of the RPE involved testing for gene expression of 91 RPE or AMD- and inflammation-relevant genes, including genes for VEGF-A and its receptors, as well as interleukin 6, interleukin 8, complement factor H and different toll-like receptors, RPE65 as well as different molecules important for antioxidative defense mechanisms (refer Appendix A for a complete list). After mid-term stimulation and comparison to untreated RNA of a parallel RPE culture, no tested gene was significantly up- or down-regulated. This strongly indicates that the Fuc1 LH fucoidan does not interfere with gene expression of unchallenged RPE cells. It has to be noted that this does not exclude effects on the protein level, because other studies showed that fucoidans can interfere with VEGF protein expression and secretion [10,11,13,14,15,20]. The lack of influence on gene expression is in accordance with a previous study focusing on fucoidan extracts of *Dictyosiphon foeniculaceus* which showed no relevant effect on VEGF-A and the VEGF receptors genes in tumor cells or APRE-19 cells [10]. However, we found a tendential reduction of the genes for CC-chemokine ligand 11, c-reactive protein, CX3C chemokine receptor 1 and leptin complementing the findings of Kopplin et al. 2018 which showed decrease of secreted proteins such as CXCL10 [5]. This suggests that Fuc1 may have anti-inflammatory, anti-complement-system-activating and lipid metabolism-relevant effects which should be further investigated, also in in vivo studies. For example, it was already shown that fucoidans can have insulin sensitivity-improving effects in mice and therefore might be useful as diabetic drugs [53]. Furthermore, they can interfere with the glucose uptake and transport in in vitro and in vivo models [54]. Further tests with Fuc1 regarding bioactivity and glucose level would be interesting, as well as for a possible treatment of diabetic retinopathy. We also could show that the secretion of proinflammatory cytokines is influenced by Fuc1 as it decreases LPS-induced IL-6 and IL-8 secretion and thereby shows anti-inflammatory properties. The reducing effect of these proinflammatory cytokines indicates an inhibiting effect on the TLR4 pathway and thereby may prevent proinflammatory downstream reactions such as activation of nuclear factor kappa-light-chain-enhancers of activated B cells. Interestingly, a different fucoidan from *Fucus vesiculosus* could activate the TLR4- mediated reactions, leading to apoptosis of lung cancer cells [55]. Furthermore, fucoidans can also affect TLR2- and TLR3-mediated pathways [56,57].

In addition to the biomedical assessment, the combination of FTIR and Raman as complementary methods was used to further elucidate structural characteristics of the previously well- characterized Fuc1 sample, broadening the scope of analytical techniques applicable for fucoidan characterization.

In conclusion, the data of this study indicate negligible effects of Fuc1 on RPE function, strongly supporting its safety in ocular use. The anti-oxidative and VEGF-lowering Fuc1 showed no relevant effects on RPE cellular functions or gene expression for mid-term treatments of RPE cells. Furthermore, we could show for the first time that this fucoidan additionally displayed anti-inflammatory properties. This paves the way for further studies testing possible applications for treatment of AMD. Further research of this sustainable high-molecular weight fucoidan should be conducted in order to explore and exploit its potential in treatment and prevention of retinal diseases.

## 4. Material and Methods

### 4.1. Cell Culture and Fucoidan

The preparation of primary RPE cells from pigs’ eyes was conducted as previously established [58,59]. In brief, after removing the cornea, retina and vitreous, cells were detached with trypsin and ethylenediaminetetraacetic acid (EDTA). The cultivation medium was HyClone DMEM (GE Healthcare, Chicago, IL, USA) with addition of 1% penicillin/streptomycin (Sigma-Aldrich, St. Louis, MO, USA), 2.5% HEPES (PAN-Biotech, Aidenbach, Germany), 10% fetal calf serum (Linaris GmbH, Wertheim-Bettingen, Germany) and 1% non-essential amino acids (PAN-Biotech). Cells were incubated at 37 °C and 5% CO_2_. RPE cells were treated two weeks after preparation, after they reached confluence. The Norwegian LH fucoidan Fuc1 (1548.6 kDa) from past studies was used [5,13], provided by Alginor ASA. The extraction and solving procedure as well as chemical characteristics such as monosaccharide composition, sulfate content, molecular weight and structure was described by Dörschmann et al. 2019 [13].

### 4.2. Methyl Thiazolyl Tetrazolium Assay

A common MTT assay [60] was performed as previously described [13]. It was used for determination of cell viability after treatment of RPE cells with Fuc1 for three and seven days. In brief, 0.5 mg/mL MTT agent was added to the cells for two hours and cells were detached with DMSO. The suspension was transferred to a 96-well plate and measured at 550 nm with Elx800 (BioTek Instruments Inc., Bad Friedrichshall, Germany).

### 4.3. Scratch Assay

The scratch assay was used for determination of wound healing ability of RPE cells and was conducted as described before [15,20]. RPE cells were stimulated with Fuc1 for three days. A scratch into the confluent RPE layer was applied with a sterile pipette tip and light microscope photos of six different spots of the scratch were taken per well. The same spots were captured again 24 and 48 h post-scratch, using colored vertical lines as orientation. The pictures were evaluated with AxioVision software (Carl Zeiss AG, Oberkochen, Germany) to measure the wound area. The area was set in relation to the initial scratch in percent.

### 4.4. Western Blot

To detect the RPE65 expression, Western blots were conducted as described by Klettner et al. 2020 [61]. In brief, cell lysates (described by Dithmer et al. 2014 [20]) were made after stimulating the cells with Fuc1 for three days. Cells were lysed with NP-40 buffer with 1% Nonidet P40 (Sigma-Aldrich, St. Louis, MO, USA), 150 mM NaCl and 50 mM Tris for 45 min. Protein content was detected photometrically with the Bio-Rad protein assay (Bio-Rad Laboratories, Inc., Hercules, CA, USA). Sodium dodecyl sulphate–polyacrylamide gel electrophoresis (SDS-PAGE) and Western blot were performed as described before [61,62]. Equal protein amounts were applied in SDS-PAGE in a 12% acrylamide gel for separating. The gels were used in a wet tank blot procedure. Membranes were blocked with 4% dry milk (Carl Roth GmbH + Co. KG, Karlsruhe, Germany) in Tris buffered saline and Tween (TBST) (Merck, Darmstadt, Germany) for one hour. Primary antibody incubation was conducted with mouse anti-RPE65 (1:6000, abcam, Berlin, Germany) and rabbit anti-β-Actin (1:2000, Cell Signaling Technologies, Denver, CO, USA) overnight at 4 °C. As secondary antibodies, anti-rabbit and anti-mouse horseradish peroxidase conjugates were used, respectively (both 1:1000, Cell Signaling Technologies). The membranes were treated with ECL Western Blotting Detection Reagent (GE Healthcare, Chicago, IL, USA) and the signal was measured with MF-ChemiBis 1.6 (Biostep, Jahnsdorf, Germany). Band volumes were calculated with TotalLab software (Biostep) and normalized with β-Actin.

### 4.5. Phagocytosis Assay

The phagocytosis assay was performed as described before and photoreceptor outer segments were isolated from retinae from pig eyes [15,63]. Fluorescence-labelled latex beads were opsonized with the photoreceptor segments. Cells cultivated on collagen I-coated cover slips were treated with Fuc1 for three days and the opsonized beads were added for four hours. Hoechst was used for staining the cell nuclei. The intracellular green beads and the blue cell nuclei were captured with a fluorescence microscope (Zeiss) and evaluated with AxioVision (Zeiss) and Fiji Software (ImageJ2, open-source software, version 2.9.0, National Institutes of Health, Bethesda, MD, USA). Cell nuclei were counted per hand and beads were counted automatically with a self-programmed macro of Fiji. Numbers were set in relation to calculate phagocyted beads per cell.

### 4.6. RPE/Choroid Organ Cultures

The RPE/choroid cultures were prepared as previously described [20,64]. In brief, pig eyes were freed from adjunct tissue and sterilized in iodide solution. The anterior part of the eye was cut and RPE/choroid tissue was removed by micro scissors and clamped into fixation rings. Six intact tissue sheets were put into one perfusion chamber (Minucells & Minutissue, Bad Abbach, Germany). The chamber was put on a heating plate at 37 °C, and a constant media flow of 2 mL/h was implemented with DMEM/F12 (1:1) with 1% penicillin/streptomycin, 2.5 mM HEPES, 110 mg/mL sodium pyruvate (all PAN-Biotech) and 10% fetal calf serum (Linaris GmbH). Gas and pH were set in balance by the silicon tubes and HEPES. Two days after preparation the chambers were stimulated with 50 µg/mL Fuc1 or same volume of media (control) and simultaneously, 50 µg/mL Fuc1 was also given to the media which flowed into the stimulated chamber. Before stimulation as well as six, 24, 48 and 72 h after, samples were taken of the flow from the chambers by collecting them for one hour. These were centrifugated for 5 min at 13,000 rpm and the supernatant was analyzed with porcine VEGF Quantikine ELISA (R&Dsystems, Minneapolis, MN, USA) as described by the manufacturer. The tissue was removed from the rings and stained with 10 µM calcein-AM (acetoxymethyl; AnaSpec, Fremont, CA, USA) for 30 min at 37 °C. The tissue was washed two times with PBS and fluorescence images were taken with an AxioVert 160 microscope (Absorbance 494 nm, Emission 517 nm).

### 4.7. Real-Time PCR

After stimulating RPE cells with 50 µg/mL Fuc1 for three days (and in parallel just changing media for an untreated control), the RNA was isolated with NucleoSpin RNA Kit and digested with DNase (both from Macherey-Nagel, Düren, Germany) as described in the user manual. cDNA was generated with High-Capacity cDNA Reverse Transcription Kit from Thermo Fisher Scientific (Waltham, MA, USA) as described in the user manual. Quantitative real-time PCR was performed using TaqMan™ gene arrays. They included 91 different primers for porcine genes (list of genes in Appendix A) with dye label 5(6)-carboxyfluorescein-minor groove binder (FAM-MGB). Real-time PCR was conducted as described in the manual of TaqMan™ Fast Advanced Master Mix from Thermo Fisher Scientific. *GAPDH*, *GUSB* and *ACTB* were used for normalization. Data evaluation was performed in Thermo Fisher Connect Cloud Software (Relative Quantification). The ∆∆CT values were used to determine the relative gene expression compared to the untreated control genes [65].

### 4.8. Inflammation Assays

For inflammation-relevant testing, confluent RPE cells were stimulated with 1, 10, 50 and 100 µg/mL Fuc1 and/or 1 µg/mL LPS for seven days. After four days, media with agents was exchanged and the stimulation continued until day 7. After this, three-days- old supernatants were collected, centrifugated at 13,000 rpm for five minutes and the supernatant used for porcine IL-8/CXCL8, IL-6 and TNF-alpha Quantikine ELISA Kits (R&Dsystems) as described by the manufacturer.

### 4.9. Vibrational Spectroscopy—FTIR and Raman

FTIR spectroscopy was performed using a Bruker Alpha II, equipped with a Platinum ATR module, a Rocksolid^TM^ interferometer and a deuterated triglycine sulphate (DTGS) detector. About 10 mg of the purified fucoidan was pressed into a small tablet (no KBr used). The sample was scanned from 400–4000 cm^−1^ (integration time of 360 s). The OPUS software was used to process the data.

Raman micro spectroscopy was performed at room temperature using a Bruker Senterra II Spectrometer equipped with a 785 nm laser. The purified fucoidan sample was scanned from 55–3650 cm^−1^ (integration time 15 s, two accumulations). A sample size of less than 1.0 mg gave sufficient resolution due to the microscope array. No further sample preparation was necessary.

### 4.10. Statistics

At least three independent experiments were done per assay. For statistics, Microsoft Excel 2010 (Microsoft, Redmond, WA, USA) as well as GraphPad PRISM 9 (GraphPad Software, Inc., San Diego, CA, USA) were used. All data in this study were checked for normality with the Shapiro–Wilk test and were normally distributed. For calculation of significances, analysis of variance (ANOVA) with post-hoc Dunnett’s multiple comparison test was performed. Real-time PCR data were evaluated with Thermo Fisher Connect with the integrated Student’s *t*-test. Results were considered as significant if *p* ≤ 0.05. Diagram bars and attached lines depict the mean and standard deviation, respectively.

## 5. Conclusions

The goal of this study was to further investigate the high-molecular weight LH fucoidan Fuc1, after showing its VEGF-inhibiting and protective effects against oxidative stress in ocular cells in a previous study. This time, the focus was its potential impact on typical RPE functions, as well as on gene expression, inflammatory activities and VEGF secretion in organ cultures. This involved testing of mid-term influence on morphology, cell viability, wound healing ability, phagocytic ability, RPE65 expression, IL-6 and IL-8 secretion, VEGF secretion in RPE/choroid cultures and gene expression of 91 different porcine RPE-relevant genes. Cell viability and morphology after seven days of fucoidan treatment were not affected, confirming its non-toxic properties. Wound healing was slightly slowed down 48 h after scratch application, but only for 10 µg/mL Fuc1, which could be a transient irrelevant effect. Phagocytosis and RPE65 expression were not significantly influenced, indicating that this marine substance does not interfere with phagocytosis and visual pigment recycling. Fuc1 was able to decrease IL-6 and IL-8 secretion of proinflammatory activated RPE cells as well as VEGF secretion of RPE/choroid cultures. With this we confirmed the anti-VEGF effects in organ cultures and also showed for the first-time anti-inflammatory activities. Furthermore, no relevant change in gene expression of any of the 91 tested genes could be determined, confirming again its safety regarding RPE functions. The results of this study render Fuc1 as a promising substance for further research concerning ocular diseases as it combines antioxidative, VEGF-inhibiting and anti-inflammatory properties with a negligible effect on RPE function. With this we pave the way for further AMD-relevant research in animal models.

## Figures and Tables

**Table 1:** Assignment of the chemical groups of Fuc1 through Raman and Fourier-transform infrared spectroscopy. The δ and ν refer to bending and stretching vibrations, τ refers to torsion vibrations, and *s* to symmetrical and *as* to antisymmetrical modes.

Wavenumber [cm^−1^]	Assignment	Activity
576	δ(pyranoid ring C-C-O)	Infrared
586	δ_s_(pyranoid ring C-C-O)	Raman
680	δ(pyranoid ring C-O-C)	Infrared
824 (shoulder)	δ(2C-O-S)	Raman
838	ν(α-pyranoid ring C-C) + ν(2C-O-S)	Infrared
839	δ(4C-O-S)	Raman
911	τ(CH_2_) + δ(2C-O-S)	Infrared
961	ν(C-O in C-O-C) + δ(C-O-H)	Infrared
994	ν_s_(C-O-S) + ν(glycosidic C-O)	Raman
1030	δ(C-O-H) + ν(C-C) + ν(C-O-S)	Infrared
1066	ν_s_(O=S=O) + ν(2C-O-S)	Raman
1078	ν(C-O) + ν(C-C) + δ(C-O-H)	Infrared
1128	ν(C-O-C) + ν(C-O-S)	Infrared
1162	ν(C-O)	Infrared
~970–1200	ν(pyranoid ring C-C) + ν(C-O)	Raman
1217	ν(S=O)	Infrared
1257 (shoulder)	ν(S=O)	Infrared
1273	ν_as_(O=S=O)	Raman
1336	ν(C-O) + δ(C-O-H)	Raman
1387	δ_s_(CH_3_)	Infrared
1454	δ_s_(CH_3_)	Raman
1636	ν(C=O) + ν(C=C)	Infrared
~1650–1800	ν(C=O)	Raman
2947	ν(C-H)	Raman
3409	ν(O-H)	Infrared

## Data Availability

Data is available on request.

## Appendix A

**Table A1:** List of genes used for real-time PCR.

Gene Symbol	Assay ID	RQ	*p*-Value
*18S-RNA*	Hs99999901_s1	0.903	0.868
*ABCA1*	Ss04955209_m1	1.799	0.394
*ABCA4*	Ss06884373_m1	0.69	0.507
*ACE*	Ss04953780_g1	1.276	0.78
*ACTB*	Ss03376081_u1	-	-
*ALB*	Ss03378640_u1	1.062	0.949
*ANXA5*	Ss06880508_m1	1.196	0.405
*APOE*	Ss03394681_m1	0.855	0.381
*B2M*	Ss03391154_m1	1.34	0.538
*BDNF*	Ss03822335_s1	1.132	0.731
*C2*	Ss03389255_m1	1.076	0.636
*C3*	Ss03391255_m1	2.036	0.566
*C5*	Ss03391586_m1	1.069	0.832
*C9*	Ss03388866_m1	1.13	0.868
*CCL11*	Ss03377222_u1	0.615	0.089
*CCL19*	Ss04246386_m1	0.868	0.83
*CCL2*	Ss03394377_m1	1.182	0.839
*CD46*	Ss03392461_u1	1.056	0.449
*CD55*	Ss03392383_m1	0.935	0.93
*CD59*	Ss03394252_m1	1.063	0.49
*CFB*	Ss03389385_g1	1.028	0.956
*CFH*	Ss03391439_m1	0.9	0.795
*CFI*	Ss06935384_m1	0.872	0.684
*CLU*	Ss03391130_m1	1.013	0.928
*COL14A1*	Ss06865093_m1	1.268	0.801
*CP*	Ss04330806_m1	0.842	0.697
*CRP*	Ss03390889_m1	0.615	0.089
*CRYA1*	Ss06837084_m1	0.596	0.544
*CRYAB*	Ss06921086_m1	1.197	0.701
*CST3*	Ss03388477_m1	0.867	0.576
*CTSD*	Ss03379762_u1	1.068	0.735
*CX3CR1*	Ss06883230_m1	0.615	0.089
*CXCL12*	Ss03391855_m1	0.81	0.892
*DICER1*	Ss04248150_m1	1.129	0.352
*EFEMP1*	Ss04326723_m1	0.924	0.853
*ELN*	Ss04955056_m1	0.401	0.241
*ERCC6*	Ss06934989_m1	1.345	0.408
*FANCG*	Ss06877694_g1	1.075	0.766
*FASLG*	Ss03381579_u1	0.564	0.54
*FBLN5*	Ss04805024_m1	0.977	0.947
*FLT1*	Ss03375679_u1	0.939	0.832
*FN1*	Ss03373673_m1	0.208	0.416
*GAPDH*	Ss03375629_u1	-	-
*GFAP*	Ss03373547_m1	0.378	0.445
*GUSB*	Ss03387751_u1	-	-
*HIF1A*	Ss03390447_m1	1.344	0.348
*HMOX1*	Ss03378516_u1	1.029	0.941
*HO2*	Ss03376410_u1	0.936	0.758
*HTRA1*	Ss06876775_m1	0.214	0.43
*ICAM-1*	Ss03392385_m1	1.135	0.83
*ICA*	Ss04955454_m1	0.727	0.47
*IGF1*	Ss03394499_m1	0.847	0.84
*IL6*	Ss07308316_g1	0.367	0.477
*IL8*	Ss03392437_m1	2.768	0.438
*KDR*	Ss03375683_u1	0.722	0.734
*LEP*	Ss03392404_m1	0.615	0.089
*MAPK1*	Ss04248225_m1	1.275	0.579
*MAPK3*	Ss06887442_m1	13.062	0.389
*MMP2*	Ss03394318_m1	0.968	0.884
*MMP9*	Ss03392100_m1	0.47	0.503
*NFE2L2*	Ss06886076_m1	1.169	0.214
*NKAP*	Ss04322419_m1	1.138	0.603
*NOS1*	Ss06838170_m1	0.685	0.159
*NOS2*	Ss03374608_u1	0.753	0.412
*NOS3*	Ss03383840_u1	0.885	0.627
*NOX4*	Ss06909549_m1	0.84	0.545
*PES1*	Ss04327334_m1	8.057	0.412
*PGK1*	Ss03389144_m1	1.328	0.118
*PLG*	Ss03380545_u1	0.865	0.815
*PTGS2*	Ss03394694_m1	1.261	0.792
*RHO*	Ss03394397_m1	0.893	0.962
*RLBP1*	Ss06905574_m1	0.676	0.568
*RPE65*	Ss06891920_m1	0.822	0.781
*SAG*	Ss03382439_u1	0.772	0.881
*SCARB1*	Ss03391104_m1	1.035	0.757
*SERPINE1*	Ss03392656_u1	0.179	0.436
*SERPINF1*	Ss03385090_u1	0.687	0.481
*SERPING1*	Ss03387977_u1	0.862	0.698
*SOD2*	Ss03374828_m1	1.306	0.214
*SPARC*	Ss03392006_m1	0.95	0.87
*TGFB1*	Ss04955543_m1	0.974	0.943
*TIMP1*	Ss03381944_u1	1.144	0.664
*TIMP-3*	Ss03375447_u1	0.851	0.719
*TLR3*	Ss03388862_m1	1.651	0.572
*TLR4*	Ss04956023_s1	0.632	0.653
*UBC*	Ss03374343_g1	1.038	0.993
*VCAM1*	Ss03390912_m1	1.445	0.693
*VEGFA*	Ss03393990_m1	1.226	0.418
*VIM*	Ss04330801_gH	0.245	0.496
*VLDLR*	Ss03374049_m1	1.131	0.434
*VTN*	Ss03382603_u1	0.772	0.506
*VWF*	Ss04322692_m1	0.954	0.926
